# Investigating Fungi-Derived Bioactive Molecules as Inhibitor of the SARS Coronavirus Papain Like Protease: Computational Based Study

**DOI:** 10.3389/fmed.2021.752095

**Published:** 2021-10-21

**Authors:** Aweke Mulu Belachew, Asheber Feyisa, Seid Belay Mohamed, Jerusalem Fekadu W/Mariam

**Affiliations:** ^1^College of Applied Science, Addis Ababa Science and Technology University, Addis Ababa, Ethiopia; ^2^College of Natural and Social Science, Addis Ababa Science and Technology University, Addis Ababa, Ethiopia; ^3^College of Natural and Computational Science, Addis Ababa University, Addis Ababa, Ethiopia

**Keywords:** Gromacs, binding affinity, inhibitor, auto-dock, protease, COVID-19

## Abstract

Due to the rapid growth of the COVID-19 pandemic and its outcomes, developing a remedy to fight the predicament is critical. So far, it has infected more than 214,468,601 million people and caused the death of 4,470,969 million people according to the August 27, 2021, World Health Organization's (WHO) report. Several studies have been published on both computational and wet-lab approaches to develop antivirals for COVID-19, although there has been no success yet. However, the wet-lab approach is laborious, expensive, and time-consuming, and computational techniques have screened the activity of bioactive compounds from different sources with less effort and cost. For this investigation, we screened the binding affinity of fungi-derived bioactive molecules toward the SARS coronavirus papain-like protease (PLpro) by using computational approaches. Studies showed that protease inhibitors can be very effective in controlling virus-induced infections. Additionally, fungi represent a vast source of bioactive molecules, which could be potentially used for antiviral therapy. Fifty fungi-derived bioactive compounds were investigated concerning SARS-CoV-2 PLpro by using Auto Dock 4.2.1, Gromacs 2018. 2, ADMET, Swiss-ADME, FAF-Drugs 4.023, pKCSM, and UCLA-DOE server. From the list of the screened bioactive compounds, Dihydroaltersolanol C, Anthraquinone, Nigbeauvin A, and Catechin were selected with the Auto-Dock results of −8.68, −7.52, −10.46, and −10.58 Kcal/mol, respectively, based on their binding affinity compared to the reference drug. We presented the drug likeliness, toxicity, carcinogenicity, and mutagenicity of all compounds using ADMET analysis. They interacted with the amino acid residues, Gly163, Trp106, Ser111, Asp164, and Cys270, through hydrogen bonds. The root-mean-square deviation (RMSD), root-mean-square fluctuations (RMSF), solvent-accessible surface area (SASA), and radius of gyration (Rg) values revealed a stable interaction. From the overall analyses, we can conclude that Dihydroaltersolanol C, Anthraquinone, Nigbeauvin A, and Catechin are classified as promising candidates for PLpro, thus potentially useful in developing a medicine for COVID-19.

## Introduction

The SARS-CoV-2 virus causes a lethal infection of the respiratory system. Most people infected with the virus showed mild to severe respiratory illness, while others were able to recover without demanding treatment (1). In contrast, older people and people with non-communicable diseases such as cardiovascular disease, diabetes, chronic respiratory disease, and cancer are more likely to develop severe illness and even death. Till now, the only best ways to prevent and slow the transmission of COVID-19 are to keep rooms well-ventilated, wash hands or use alcohol-based sanitizers frequently, cough into a bent elbow, practice social distancing, avoid crowds, and use face masks (2). Recently, SARS-CoV-2 mutated over time and developed new properties like vast and quick spread, disease severity, or inactive to vaccines and medicines, hence, there is a need for new diagnostic tools and other public health and social measures. Globally, several mutant viruses have already emerged and widely circulated among humans since the beginning of the pandemic. Until now, new SARS-CoV-2 variants of concern in the world including, Alpha (the United Kingdom, September 2020), Beta (South Africa, May 2020), Gamma (Brazil, November 2020), and Delta (India October 2020), Eta (Multiple countries, December 2020), Iota (the United States of America, November 2020), Kappa (India, October 2020), and Lambda (Peru, December 2020) have been reported (2–4). The COVID-19 pandemic has infected 216,867,420 million people, caused 4,507,837 million deaths, and 5,019,907,027 people have been vaccinated as of August 30, 2021 (2). In Ethiopia, from January 3, 2020, to August 31, 2021, there have been 306,810 confirmed cases of COVID-19 with 4,660 deaths and 2,434,041 people vaccinated, according to the report of WHO. Even though safe and effective vaccines are available to protect people from getting COVID-19, there is no effective inhibitor for the SARS-CoV-2 protease to prevent people from being seriously ill or dying (1, 2, 4). At present, computer-aided drug designs have been used as an efficient alternative for recognizing reliable candidates that can be repurposed drugs or/and phytochemicals to treat viral infection, including COVID-19 (5–7). Papain-like protease (PLpro) is an essential coronavirus protein required for the processing of viral polyproteins to generate functional protein for virus replication and enable viral spread; therefore, the inhibition of the PLpro is a feasible strategy to develop antiviral drugs and suppress the ongoing SARS-CoV-2 impacts (3–5). Furthermore, they are unique to the different viruses including COVID-19, thus offering the potential for specific treatments that produce minimal toxic side effects (5). Therefore, SARS-CoV-2 PLpro makes it an attractive antiviral drug target but, there is a limited study to address this area. Furthermore, studies revealed that PLpro and chymotrypsin-like protease (3Clpro), aka the main protease, are druggable targets (8, 9). Therefore, we screened the interaction of a library of fungi-derived bioactive molecules against PLpro as inhibitors with binding pocket residues.

For a long time, bioactive compounds isolated from microorganisms, plants, or animal sources have been utilized to treat infectious and non-infectious diseases (10–12). Several studies revealed that fungi provide different bioactive compounds with diverse activities and are even developed into drugs such as Cyclosporine, Caspofungin, Lovastatin, and Fingolimod (13–15). Bioactive compounds with potent antiviral activity are presently under investigation, and the number of studies is continually increasing (14–16). Up until now, hundreds of fungi have been investigated for their metabolites, and most of them have been proven to be rich in bioactive compounds. Moreover, several novels and valuable bioactive compounds with antiviral, antimicrobial, insecticidal, cytotoxic, and anticancer activities have been reported from fungi (14, 15, 17–19). Previously, the potentials of fungal metabolites as anti-viral agents were explored and the success was promising. As far as antiviral therapy is concerned, the fungi metabolites helped treat viral infections, such as AIDS, influenza, and hepatitis, the leading causes of human death worldwide, and the metabolites belonged to the chemical class of Indole alkaloids, non-ribosomal peptides, polyketides, and terpenoids (14, 16, 19). With the urgent need for safe and effective drugs to treat COVID-19, we have explored bioactive molecules isolated from fungi that have been reported to possess an anti-HIV protease (13–16, 18–20). Conceptually, an identical work on fungi secondary metabolites, utilizing a similar method, was proposed by Fu et al. (7) targeting PLpro. With this rationale, the fungal metabolites were looked for in pursuing this research. Still, the negative role of the COVID-19 virus has increased, and new types of variants have emerged. Hence, the problem of the development of new antivirals has remained a big challenge. This makes it possible for candidates to develop more effective drugs from fungi, affecting different stages of virus reproduction and inhibiting the pathogenesis of the disease. The discovery and characterization of fungi metabolites due to their vast diversity, stereochemical properties, and preapproved biocompatibility having antiviral activities is an emerging field of research, and several compounds have been identified as promising candidates for new drug discovery (20–23). This study aims to investigate the binding affinity and stability of fungus-derived bioactive molecules and provide an insight into the therapeutics that might help treat COVID-19. In this study, 50 molecules were screened based on their binding energy and hydrogen bonds to PLpro via molecular docking, ADMET, and molecular dynamics study for 100 ns.

## Materials and Methods

### The Platform for Molecular Modeling

A server computer with specifications processor (CPU) Intel® Xeon E5110 (Intel, Santa Clara, California, USA), graphics processing unit (GPU) Nvidia® GeForce GTX 780 (Nvidia, Santa Clara, California, USA), and 32GB Random Access Memory (RAM) DDR2 with Linux Ubuntu 20.04 LTS (Linux, San Francisco, California, USA) was used. Molecular dynamics (MD) simulations were performed by GROningen MAchine for Chemical Simulations (GROMACS 2018.2).

### Bioactive Compounds Preparation

After conducting a literature review, 50 bioactive compounds were collected from different fungi through PubMed, Pub-Chem, and Google scholar platforms. Each compound was prepared to perform the molecular docking study. The statutory declaration form (SDF) of each structure was retrieved from the Pub-Chem database and presented in Supplementary Table S1 (24). The bioactive compounds were uploaded into Avogadro (AV) followed by energy minimization and optimization by using the algorithm with the steepest descent for 2,000 steps (25). Finally, the 3D structure of all the bioactive molecules was generated by adding hydrogen atoms and was saved as a program database (PDB) file for further analysis.

### Protein Preparation

The crystal structure of the SARS CoV-2 PLpro in complex with the GRL0617 ligand (PDB ID: 7CJM) was retrieved from the Protein Data Bank (26). The protein structure was prepared by using Pymol (27) and Discovery Studio software (28) and then saved as a PDB file. The missing residues and 3D protonation were conceded on the PLpro via the Swiss-PDB viewer (Swiss Institute of Bioinformatics, Switzerland) and H++ server (Department of Computer Science, Virginia Tech, Blacksburg, VA, USA). During the preparation, the protein bond orders were assigned and hydrogen atoms were added as well. Water molecules and every heteroatom in the protein structure were removed. The cleaned crystal structure was then optimized, verified, and energy-minimized using the GROMOS 43B1 force field via the Swiss-PDB viewer (29). Finally, the modeled structure was validated using the UCLA-DOE server (http://servicesn.mbi.ucla.edu/) as discussed in our previous study (30). The modeled 3D structure was then confirmed by using the RAMPAGE, ERRAT, and Verify 3D online servers. After, validating the structure, we resolved the issue of the mismatched residues and missed structure across the models.

## Docking Methodology

The main focus of the experiments was to calculate the binding affinity with Auto-Dock 4.2 according to the previous study procedure (27). In this experiment, 50 bioactive molecules were screened for the binding affinity with PLpro binding sites. Before each docking experiment, the SARS CoV-2 PLpro structure was first prepared in Auto-Dock. First, the water molecules and the original inhibitor were removed from the PLpro structure. Then, any missing atoms were added. The optimization step was then employed to provide stable conformation before converting to PDBQT format for the docking analysis. All ligands and PLpro structures were converted to PDBQT format to prepare them in an acceptable format for docking. To cover the whole protein, structure global docking was conducted with the spacing of 0.5 Å. The grid box was set to 126 × 108 × 114 points in an *xyz*-dimension that equated to a grid box spacing of 0.5 Å^3^, and the coordinate of the *x, y*, and *z* centers of the box was at 0.214, 15.986, and 14.518, respectively. All the docking parameters procedures were set to 250 genetic algorithm runs using the Lamarckian genetic algorithm conformational search, with the population size of 2,500,000 maximum numbers of energy evaluations, and 300 generations per run. Lastly, the best SARS CoV-2 PLpro-fungi derived bioactive complexes were selected according to the molecular docking results including binding energy, root mean square deviation, and type of favorable interactions and binding sites for further analysis.

### *In silico* Drug Likeness and Toxicity Assessment

The structures of the compounds presented in the fungi-derived bioactive compounds (Supplementary Table S1) were obtained from the Pub-Chem database in the simplified molecular input line entry specification (SMILES) files and used for the webservers to generate, predicting their drug-likeness properties. Then toxicity assessment was made with online tools such as SwissADME (SIB Swiss Institute of Bioinformatics, 1015 Lausanne, Switzerland); FAF-Drugs 4.023 (MTi molecule Therapeutiques in silico, France) and pKCSM (Bio21 Institute University of Melbourne, 30 Flemington Rd - Parkville, Melbourne, VIC 3052, Australia) (31–33). Furthermore, the environmental toxicity assessments were also evaluated through admetSAR24 (32). The drug-likeness of the selected bioactive compounds was evaluated through the Lipinski Rule of Five to predict their pharmacokinetic properties using the Swiss ADME server (31).

### Molecular Dynamic Simulations

A molecular dynamic simulation of the top three docked complexes was run to see the structural stability by using a Gromacs package (2018.2) for 100 ns, in which the AMBER14 force field (34) was employed. A cubic simulation box was created and the PLpro-fungi-derived bioactive molecules complex was placed in with TIP3P water molecules. And then, the simulation environment was neutralized at a temperature of 299 K with sodium chloride (NaCl). The simulation box was extended at 1 Å more than the PLpro-fungi-derived bioactive molecules complex, for them to be able to move freely. The long-range electrostatic interactions were calculated using Particle Mesh Ewald's algorithms by setting a cutoff radius of 3.5 Å (35). After these steps, the NVT [amount of substance (N), volume (V) and temperature (T) are conserved] stage was carried out to minimize the system structure and simultaneously, followed by the NPT [amount of substance (N), pressure (P) and temperature (T) are conserved] stage for the equilibration of the system structure by using 1,000 ps simulation time at the constant temperature of 298 K and pressure of 1 bar. The production part was carried out for 100 ns with the time step of 2 fs. Pymol and VMD 1.9.1 (36) were used to visualize the trajectory. Lastly, data analyses were performed for each complex and free PLpro by using root-mean-square deviation (RMSD), root-mean-square fluctuation (RMSF), the radius of gyration (Rg), and solvent-accessible surface area (SASA).

## Discussion

**Figure 1:**
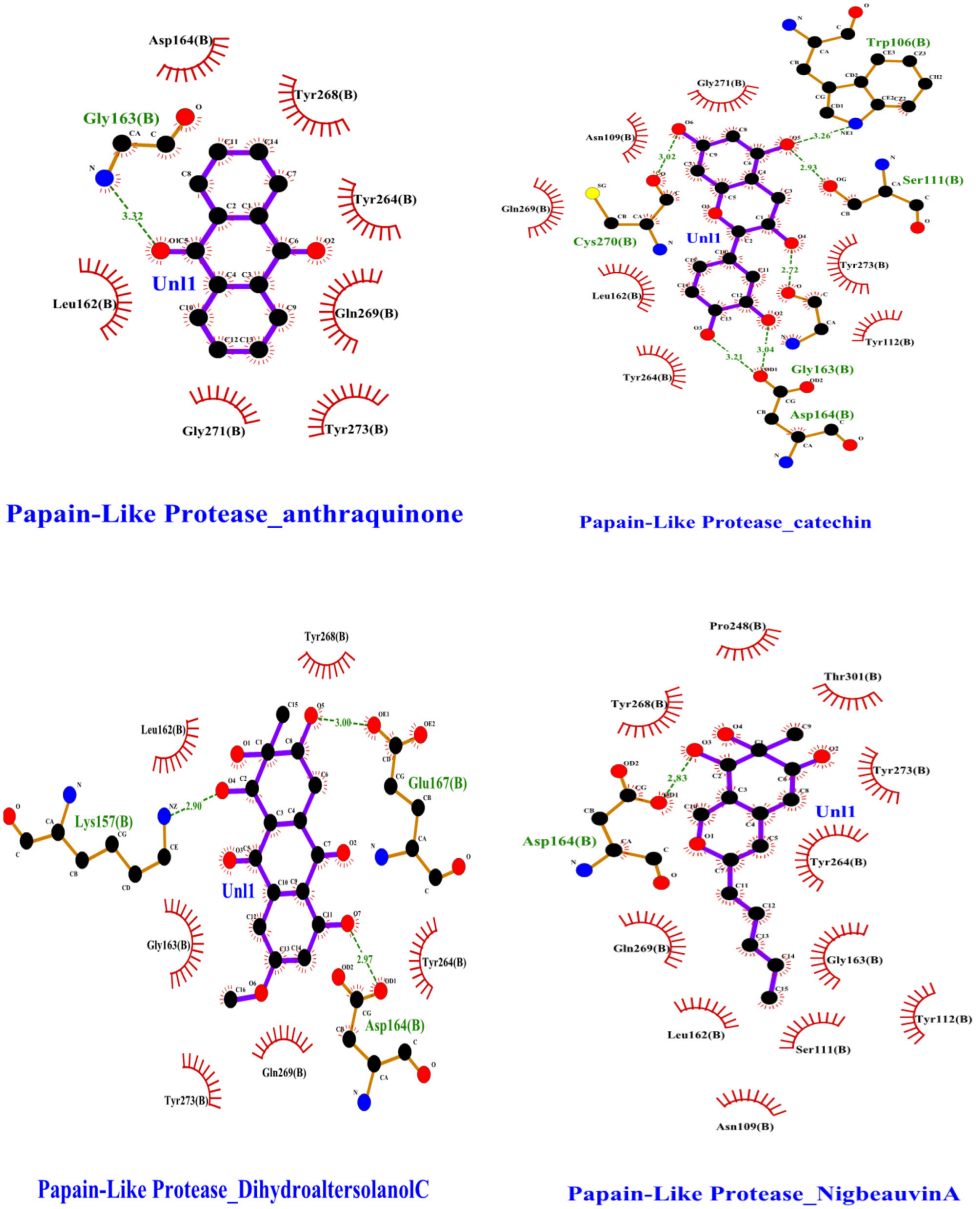
Interactions of the selected bioactive molecules and SARS-CoV-2 Papain-Like Protease (Plpro) binding pocket residues. For all the bioactive molecules, carbon atoms are shown in black, oxygen in red, and nitrogen in blue. Bonds in the Anthraquinone, Dihydroaltersolanol C, Catechin, and Nigbeauvin A.

To find a potential candidate for treating COVID-19 from bioactive fungus-derived molecules, first, we performed molecular docking studies on 50 molecules toward the binding pocket of the enzyme COVID-19 (PDB ID: 7CJM) (7). The minimized structure of the inhibitor, GRL0617, was re-docked into the original binding site of the PLpro. The results of the 150 Genetic Algorithm runs of the re-docking were ranked and shown in Table 1. The best docking score of GRL0617 was −7.85 kcal/mol. The superimposing of the re-docked PLpro-GRL0617 over the original X-ray structure indicated that GRL0617 was able to seek out the location of the binding site. For this study, the list of the bioactive fungus-derived molecules tested for the docking study is revealed in Supplementary Table S1. Out of the screened bioactive agents against the target, the PLpro enzyme was reported at the top four, based on their docked score. Accordingly, all the bioactive compounds reported here showed docking scores higher than −7.4 kcal mol−1 which are higher than the reference drug (Table 1). The computational screening technique offered a reasonable screening result from an array of drugs and phytochemicals as a credible inhibitor for target molecules (5–7). But, the protein folding reactions took place at an ms level, which was at the limit of the accessible simulation times. It is still difficult to simulate the whole process of protein folding using the conventional MD method. Another limitation is that the study entirely focused on computational investigation. Based on our molecular docking results, four potent inhibitors targeting the SARS-CoV-2 PLpro were identified to exhibit significant binding affinities and interaction with the active site and pocket site, which are vital for inhibition as reported with the reference drug. Our first compound, Catechin found the docking result with a low RMSD value of 24.709 Å and a binding affinity score of −10.58 kcal/mol^−1^ (Table 1). The second compound, Nigbeauvin A found the docking result with a low RMSD value of 25.564 Å and a binding affinity score of −10.46 kcal/mol^−1^ (Table 1). The third compound, Dihydroaltersolanol C was found the docking result with a low RMSD value of 21.309 Å and a binding affinity score of −8.68 kcal/mol^−1^ (Table 1). The fourth compound was found the docking result with a low RMSD value of 16.36 Å and a binding affinity score of −7.8 kcal mol^−1^ (Table 1). The Auto-Dock binding affinity results from the drugs through phytochemical and microbial studies were revealed to be within the range of −7.8 to −6.5 kcal/mol^−1^ (37–39). With the standard antiviruses repurposed drugs, the binding affinity ranges from −6.9 to −7.3 Kcal/mol^−1^ (7). Thus, Dihydroaltersolanol C, Anthraquinone, Nigbeauvin A, and Catechin showed better affinity toward PLpro and were selected for further analysis. Moreover, the 3CLpro complex formed six conventional hydrogen bonds with Ser111, Trp106, Cys270, Asp164 (2 times), and Gly163, which is consistent with the reference drug binding residues (7). In contrast, the PLpro-Nigbeauvin A complex and PLpro-Anthraquinone were stabilized by one hydrogen bond each at Asp164 and Gly163 positions, respectively, whereas Dihydroaltersolanol interacted with PLpro through three conventional hydrogen bonds at Glu167, Lys157, and Asp164 positions (Figure 1 and Table 1), which are consistent with the previous findings conducted by targeting the PLpro (7). These compounds formed numerous non-hydrogen-bonded interactions at the active site and pocket site *via* the Asn109, Ser111, Gly271, Trp106, Tyr268, Glu167, Tyr264, Leu162, Lys157, Gly163, Tyr273, Gln269, and Asp164 residues of PLpro, which are defined as binding pocket residues (7).

**Table 1:** Shows the interaction between SARS-CoV-2 Papain-Like Protease (PLpro) and the top four Fungi-derived bioactive compounds.

**Compounds**	**Binding affinity**	**RMSD**	**No. H-bonds**	**Amino acids involved in the interaction**
Reference (GRL0617)	−7.85	11.394	1	Tyr268, Gln269, Ser111, His272, Asp286, Asp164, Pro247, Pro248, Gly163, and Asp164
Dihydroaltersolanol C	−8.68	11.309	3	Tyr268, Glu167, Tyr264, Leu162, Lys157, Gly163, Tyr273, Gln269, and Asp164
Anthraquinone	−7.52	6.36	1	Leu262, Gly163, Asp164, Tyr268, Tyr264, Gln269, Gly271, and Tyr273
Catechin	−10.58	14.709	6	Asn109, Ser111, Gly271, Trp106, Tyr273, Tyr112, Gly163, Asp164, Tyr264, Leu162, Cys270, and Gln269
Nigbeauvin A	−10.46	15.564	1	Pro248, Thr301, Tyr273, Tyr264, Gly163, Tyr112, Ser111, Asn109, Leu162, Gln269, Asp164, and Tyr268

After the docking study, the top four selected bioactive molecules were screened for efficiency and safety in terms of various properties. As shown in Table 2, properties such as p-glycoprotein inhibition, carcinogenicity, hepatotoxicity, human intestinal absorption, and cytochrome P (CYP) inhibition, were predicted based on the web-based tools. Many studies showed that the toxicity and effectiveness of drugs mainly determine the success and failures of the candidate drugs in clinical trials (12, 30, 38). In the context of this study, the four top candidate bioactive molecules had no probability of toxicity, hepatotoxicity, CYP inhibition, oral toxicity, and carcinogenicity observed as shown in Table 2. To confirm the non-inhibitory effect of the selected compounds for cytochrome p450 as mentioned in the ADMET studies, we carried out docking studies against cytochrome p450 (PDB ID:6wr0), and the result revealed a high binding affinity between +6.28 to +8.59 as shown in Supplementary Table S1. The molecular weights (MWs) of the top four bioactive molecules were 322.31, 208.21, 262.30, and 290.27 g/mol for Dihydroaltersolanol C, Anthraquinone, Nigbeauvin A, and Catechin, respectively. Moreover, all the bioactive molecules were not violating Lipinski's rule of five. The numbers of the hydrogen bond donors for Dihydroaltersolanol C, Anthraquinone, Nigbeauvin A, and Catechin were reported as 4, 0, 2, and 5, respectively. The numbers of the hydrogen bond acceptors for Dihydroaltersolanol C, Anthraquinone, Nigbeauvin A, and Catechin were 7, 2, 4, and 6, respectively (Table 2).

**Table 2:** Pharmacokinetic and Toxicity Properties for the top four bioactive fungi-derived molecules, which were derived from the SwissADME, admetSAR, and pKCSM webservers.

**Parameters**	**Dihydroaltersolanol C**	**Anthraquinone**	**Nigbeauvin A**	**Catechin**
Molecular weight	322.31 g/moL	208.21 g/moL	262.30 g/moL	290.27 g/mol
H-bond acceptor	7	2	4	6
H-bond donor	4	0	2	5
CYP2D6 substrate	No	No	No	No
CYP3A4 substrate	No	No	No	No
CYP1A2 inhibitor	No	Yes	No	No
CYP2C19 inhibitor	No	Yes	No	No
CYP2C9 inhibitor	No	No	No	No
CYP2D6 inhibitor	No	No	No	No
CYP3A4 inhibitor	No	No	No	No
Carcinogenicity	Non-carcinogenic	Non-carcinogenic	Non-carcinogenic	Non-carcinogenic
Hepatotoxicity	No	No	No	No
P-glycoprotein inhibitor	No	No	No	No
Human intestinal absorption	+0.9796	+0.9956	+0.9608	+0.9887
Ames mutagenesis	−0.5000	−0.7300	−0.5900	+0.6300
Acute oral toxicity	No	No	No	No
Lipinski rule of five	Yes	Yes	Yes	Yes

**Figure 2:**
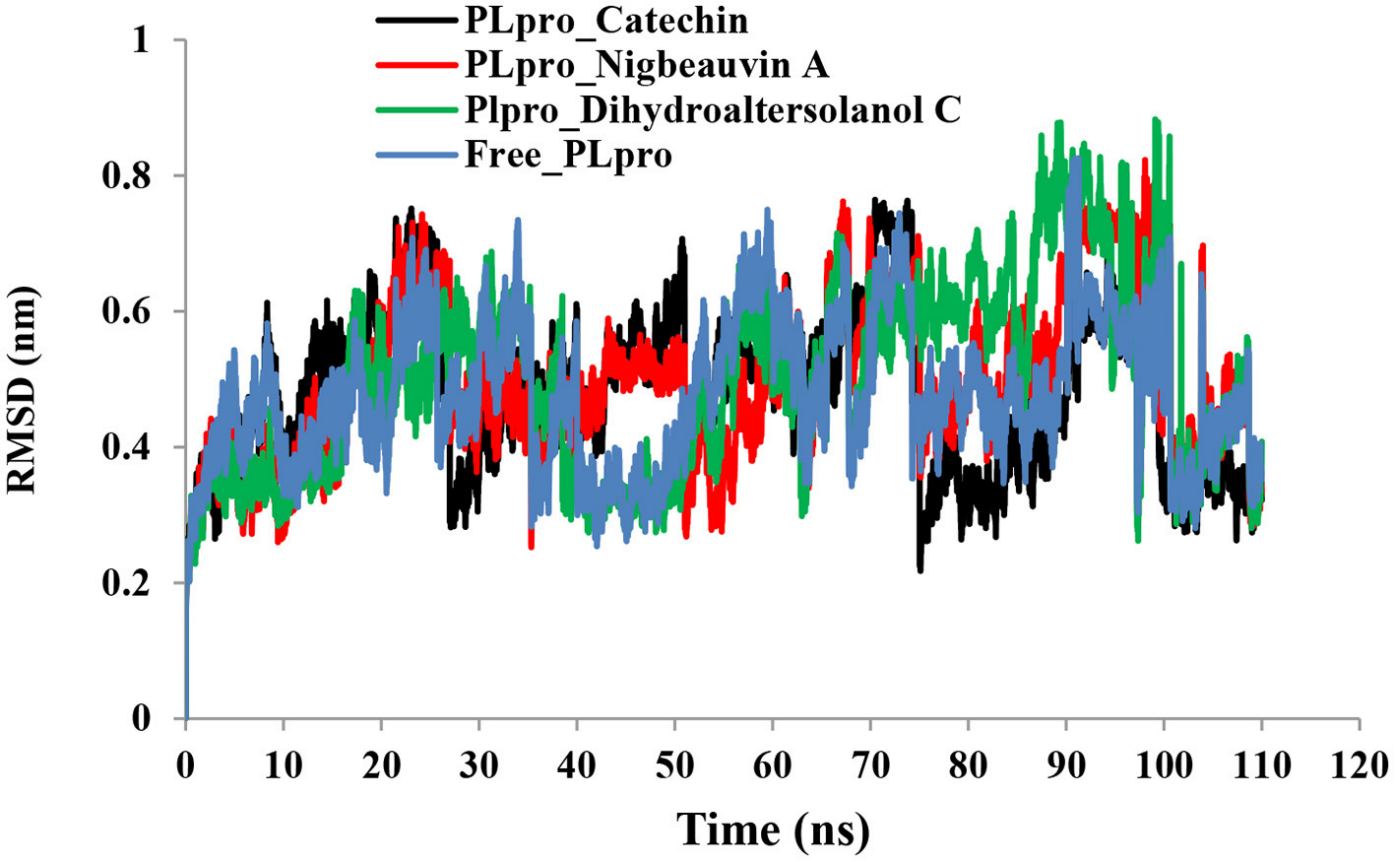
Time run root-mean-square deviation (RMSD) value analyses for the simulated PLpro-ligand complex and Free-PLpro alpha carbon atoms systems. PLpro-Catechin code: black, PLpro-Nigbeauvin A code: red, PLpro-Dihydroaltersolanol C code: green, and Free-PLpro code: blue.

**Figure 3:**
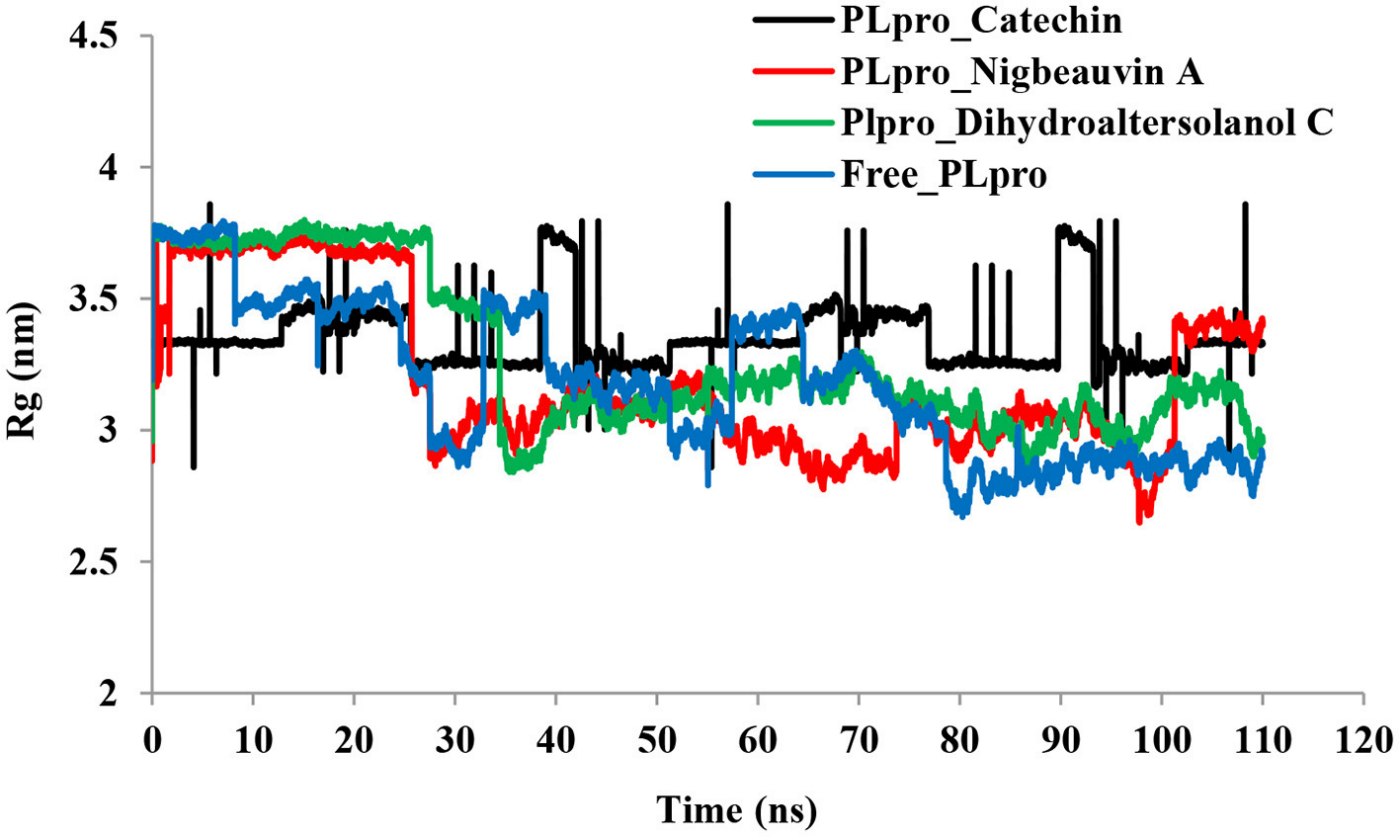
Time series for the degree of rigidity and compactness analyses for the PLpro-ligand complex and Free-PLpro backbone of atoms simulated. PLpro-Catechin code: black, PLpro-Nigbeauvin A code: red, PLpro-Dihydroaltersolanol C code: green, and Free-PLpro code: blue.

**Figure 4:**
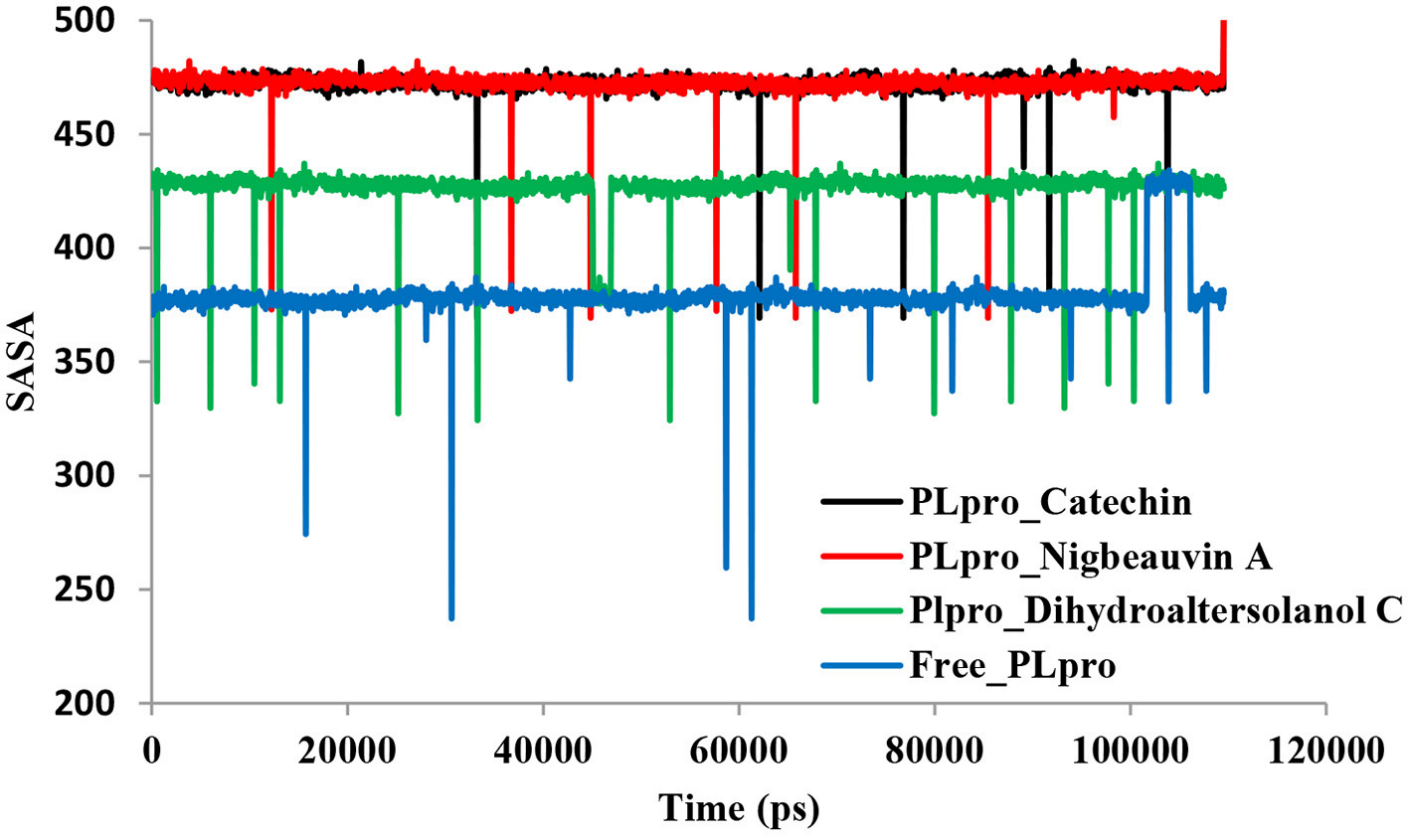
Time series for the protein volume with expansion analyses for the PLpro-ligand complex and Free-PLpro alpha carbon atoms simulated systems. PLpro-Catechin code: black, PLpro-Nigbeauvin A code: red, PLpro-Dihydroaltersolanol C code: green, and Free-PLpro code: blue.

**Figure 5:**
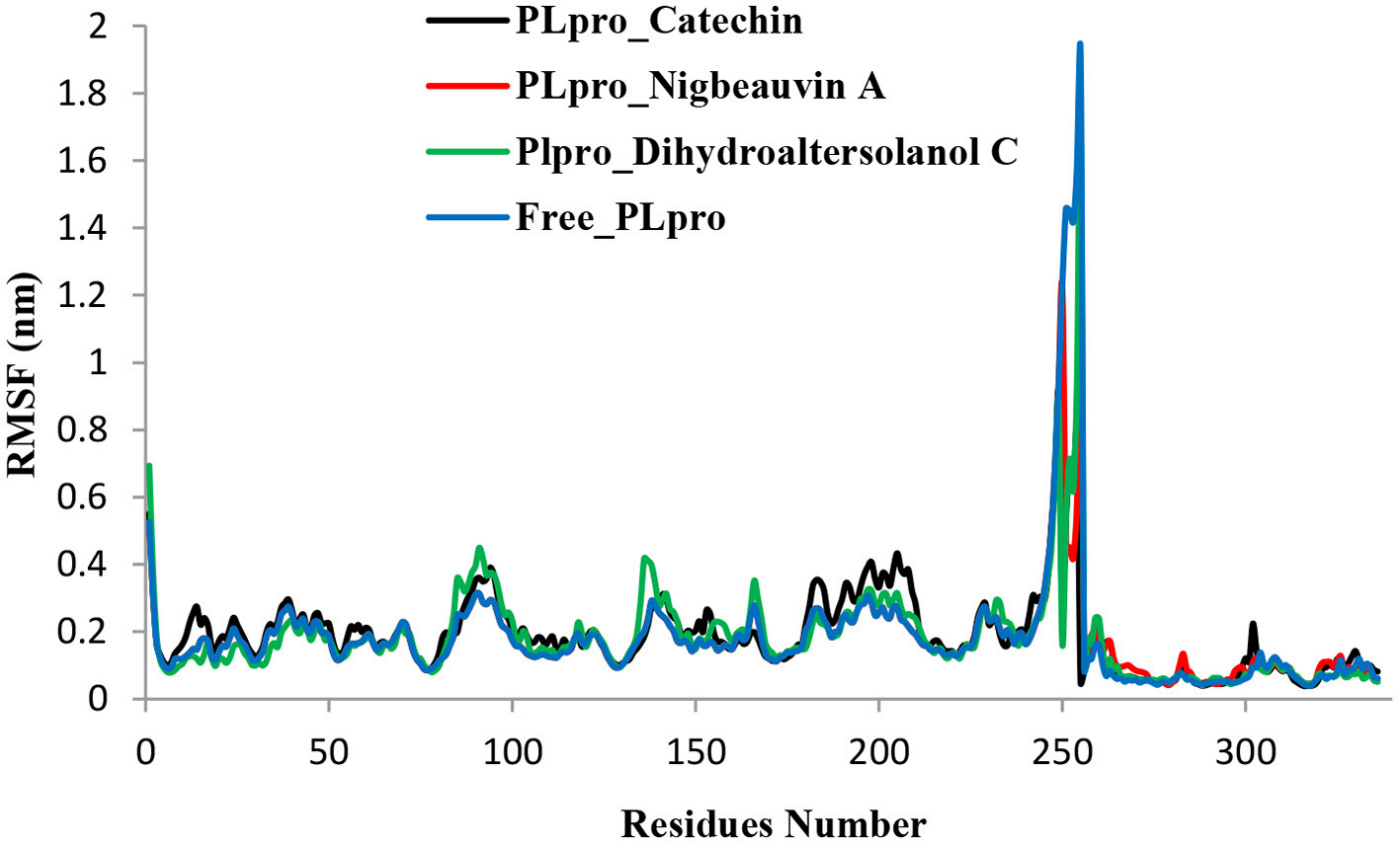
Time series for the flexibility analysis of the amino acid residues for the PLpro-ligand complex and Free-PLpro alpha carbon atom simulated systems. PLpro-Catechin code: black, PLpro-Nigbeauvin A code: red, PLpro-Dihydroaltersolanol C code: green, and Free-PLpro code: blue.

An MD simulation study was performed to support the molecular docking assessment, and identify the binding stability. First, the RMSD of the backbone atoms from the simulation trajectories were assessed to understand the changes in the three ligands (Pub-Chem CID 9064, Pub-Chem CID 132562011, and Pub-Chem CID 146684151) with PLpro as shown in Figure 2. The PLpro-Catechin complex showed an average root mean SD of 0.23–0.74 nm (Figure 2), which is consistent with the previous study (6, 16, 40). The RMSD value of the PLpro-Catechin complex revealed less stability between 20–30 ns, 40–50 ns, and 60–70 ns. This revealed that the PLpro-Catechin complex indicated maintenance of stability after 70 ns, with few fluctuations observed (Figure 2). Similarly, the other two complexes formed between PLpro-Dihydroaltersolanol C and PLpro-Nigbeauvin A shows an average root mean SD of 0.32–0.85, 0.26–0.88 nm, respectively, and both achieved stable RMSD profiles after 30 ns in the simulations. Even though, Free-PLpro exhibited a higher degree of deviation (0.24–0.88 nm) compared with PLpro-Dihydroaltersolanol C, PLpro-Nigbeauvin A, and PLpro-Catechin complex did not exceed its RMSD value more than 1 nm. The Rg values from the simulation trajectories were examined to understand the labile nature of the PLpro-Bioactive molecules as shown in Figure 3. The PLpro-Dihydroaltersolanol-C and PLpro-Nigbeauvin-A complexes decreased after 30 ns during the simulation. In contrast, PLpro-Catechin showed an increased rigidity of the simulated complexes (Figure 3). The average Rg value of the Free-PLpro, PLpro-Catechin, PLpro-Dihydroaltersolanol C, and PLpro-Nigbeauvin A complexes were 2.75, 3.5, 2.75, and 2.75 Å, respectively. Here, the PLpro without ligand and in the complex was observed to be stable as no large fluctuations were observed; the protein is stable in the complex as well, which is consistent with another finding (41). The SASA of the simulation complexes was analyzed to determine the changes in the protein volume. The PLpro-catechin and PLpro-Nigbeauvin-A complex displayed an expanded surface area throughout the simulation, which was stably maintained within the simulation environment. A lower degree of change in the SASA profile was observed for PLpro-Dihydroaltersolanol-C compared with the other two compounds, but the deviations were not larger than Free-PLpro (Figure 4). The average of the SASA from Free-PLpro, PLpro-catechin, PLpro-Nigbeauvin-A, and PLpro-Dihydroaltersolanol-C complex were 475, 475, 425, and 325 Å2, respectively. The RMSF profiles of all three compounds were analyzed to understand the changes in the amino acid residues involved in the hydrogen bond formation as shown in Figure 5. In this study, most of the residues had low RMSF values for all three complexes, except for amino acids whose value was close to 250. The binding interactions of the top three docked complexes were further evaluated after 100 ns in the simulation to understand their changes after the simulation (Table 1). The Catechin- PLpro complex formed six conventional hydrogen bonds at Ser111, Trp106, Cys270, and Asp164 (2 times), Gly163, and the RMSF values of the key residues around the active site were lower than those in the other regions of the protein, which implied that these residues had strong binding interactions with the inhibitors, similar to previous findings (7, 16, 41). The Dihydroaltersolanol interacted with PLpro through three conventional hydrogen bonds at Glu167, Lys157, and Asp164 position (Figure 1 and Table 1). Based on these results we recommended laboratory assays targeting directly various steps along virus infection are needed to understand the mechanisms of action in detail. The free PLpro RMSF analysis shows the highest fluctuation (>1.5 Å) in the regions of the amino acid at 237–265, comprising the binding site loop. The rest of the protein amino acids displayed low RMSF values highlighting their stability as well (Figure 5). The strengths of this study lie in the extensive virtual screening, molecular docking, and long molecular dynamics simulation. However, this study also suffers from some limitations such as the absence of any *in vitro* and/or *in vivo* experiments.

**Figure 6:**
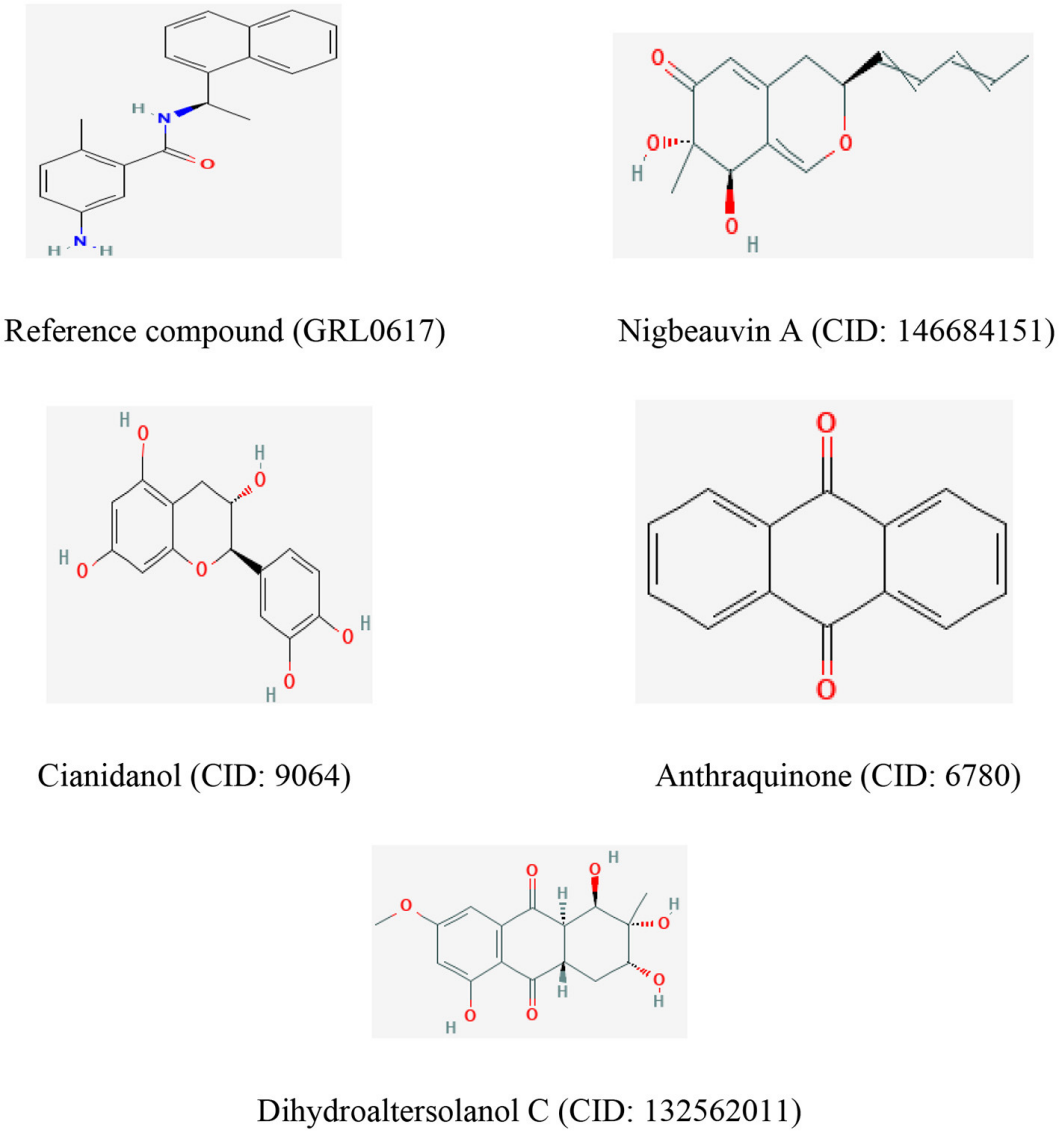
The chemical structures of the reference compound (GRL0617) and the top four selected fungi-derived compounds retrieved from Pub-Chem (https://pubchem.ncbi.nlm.nih.gov/).

## Conclusions

There has been an increased recognition that more attention needs to be paid to COVID-19 drug discovery. In recent years, the natural products of bioactive molecules have revealed significant advances in the treatment of virus infection. However, previous studies are mainly limited to a subset of phytochemicals and repurpose of the drug. There is limited study on the antiviral mechanisms of fungal products on COVID-19 infection. To overcome the shortcomings of the previous studies outlined above, we proposed 50 fungi-derived bioactive compounds and investigates their binding affinity through a computational study. In this work, the molecular docking studies identified four potent inhibitors, namely, Catechin, Nigbeauvin-A, and Dihydroaltersolanol-C out of the 50 that were identified. Moreover, the molecular simulation study of the three docked complexes supported the stable nature and rigid conformations formed by the docked complexes, as assessed by the simulation trajectories and based on multiple descriptor analyses. *In vitro* assays could be performed to confirm the precise targeting of these compounds against SARS-CoV-2. Thus, more detailed knowledge of the actual molecular targets is crucial to develop these molecules further to combat virus infections.

## Data Availability Statement

The original contributions presented in the study are included in the article/Supplementary Materials, further inquiries can be directed to the corresponding author/s.

## Author Contributions

The project approach was conceptually designed by AB. AB, AF, SM, and JF writing and original draft preparation. AB chemical structure drawing and run simulation. All authors have read and agreed to the published version of the manuscript.

## Conflict of Interest

The authors declare that the research was conducted in the absence of any commercial or financial relationships that could be construed as a potential conflict of interest.

## Publisher's Note

All claims expressed in this article are solely those of the authors and do not necessarily represent those of their affiliated organizations, or those of the publisher, the editors and the reviewers. Any product that may be evaluated in this article, or claim that may be made by its manufacturer, is not guaranteed or endorsed by the publisher.
