# Supplementary material for: Investigating Fungi-Derived Bioactive Molecules as Inhibitor of the SARS Coronavirus Papain Like Protease: Computational Based Study
Source: Front Med (Lausanne). 2021 Oct 21;8:752095. doi: 10.3389/fmed.2021.752095 (PMC8566946; doi:10.3389/fmed.2021.752095)
Supplement: Supplementary file 1 [file Data_Sheet_1.docx]

Fungi-Derived Bioactive chemical Screening by Targeting Papain-Like Protease of SARS-CoV-2 to Design Effective Potent Inhibitors: Computational study

Aweke Mulu Belachew^1*^, Asheber Feyisa^2,^ [Seid](https://peerj.com/user/143408/) Belay Mohamed^3^ and Jerusalem Fekadu W/ Mariam ^4^

^1^ College of Applied Science, Addis Ababa Science and Technology University, Addis Ababa, Ethiopia

^2 3^ College of Natural and Social science, Addis Ababa Science and Technology University, Addis Ababa, Ethiopia

**^4^** College of Computational Science, Addis Ababa Science University, Addis Ababa, Ethiopia

**Corresponding Author:**

Aweke Mulu Belachew^1*^

Addis Ababa, Ethiopia, 16417, Ethiopia

Email address: [aweke.mulu@aastu.edu.et](mailto:aweke.mulul@aastu.edu.et%20/)

**Supportive files**

**Table S1** All fungi-derived bioactive compounds and screened in this study against PLpro of SARS-CoV-2.

|  | Bioactive compounds | Binding affinity | RMSD^b^ | Pub-Chem CID |
| --- | --- | --- | --- | --- |
| 1 | Paclitaxel | -5.49 | 18.47 | 36314 |
| 2 | Baccatin III | -5.14 | 14.05 | 65366 |
| 3 | Podophyllotoxin | -4.94 | 14.20 | 10607 |
| 4 | Camptothecin | -4.88 | 14.16 | 24360 |
| 5 | Vinblastine | -4.84 | 16.41 | 13342 |
| 6 | Hypericin | -4.63 | 33.81 | 3663 |
| 7 | Huperzine A | -4.30 | 16.84 | 854026 |
| 8 | Diosgenin | -4.26 | 17.37 | 99474 |
| 9 | Toosendanin | -4.18 | 18.10 | 9851101 |
| 10 | Cis-(+)-Alpha-Irone | -1.76 | 17.43 | 5281521 |
| 11 | Hypocreolide | -4.30 | 13.71 | 102484566 |
| 12 | Cytospolide | -4.26 | 33.48 | 102134456 |
| 13 | Cytospolide D | -4.18 | 33.40 | 102134456 |
| 14 | Cytospolide E | -4.05 | 33.38 | 53307438 |
| 15 | Benzophomopsin A | -2.30 | 30.17 | 44513691 |
| 16 | Ascotricin A | -3.88 | 26.38 | 44224584 |
| 17 | Aquastatin A | -3.74 | 15.51 | 192651 |
| 18 | Dthpsad | -3.29 | 27.51 | 124202 |
| 19 | Aquastatin C | -3.25 | 29.47 | 51350336 |
| 20 | Xanthepinone | -2.87 | 29.92 | 44557222 |
| 21 | Herqueinone | -6.51 | 33.92 | 168617 |
| 22 | Myricetin | -5.52 | 33.71 | 5281672 |
| 23 | Secalonic acid D | -5.41 | 17.86 | 73431 |
| 24 | Chrysoxanthone | -5.19 | 24.23 | 101476999 |
| 25 | Desmethyldiaportinol | -5.15 | 31.46 | 24882464 |
| 26 | Cytosporone B | -5.06 | 16.10 | 10687292 |
| 27 | Ceramidastin | -5.02 | 29.34 | 42629484 |
| 28 | Emervaridione | -4.74 | 45.41 | 56933583 |
| 29 | varioxiranediol | -7.21 | 21.64 | 56933584 |
| 30 | Epicoccolide A | -6.74 | 21.65 | 71732639 |
| 31 | Epicoccolide B | -6.72 | 24.13 | 11210533 |
| 32 | Chloropestolide A | -6.31 | 24.14 | 101483980 |
| 33 | Haplofungin A | -6.29 | 21.23 | 102420396 |
| 34 | Decalpenic acid | -5.55 | 24.04 | 50925583 |
| 35 | Caripyrin | -4.95 | 27.42 | 75219706 |
| 36 | Udalactarane A | -4.27 | 16.90 | 60201877 |
| 37 | Ganodermycin | -7.16 | 19.91 | 54764417 |
| 38 | Phomopsene | -6.52 | 28.75 | 25229136 |
| 39 | Artemisinin | -6.72 | -7.52 | 68827 |
| 40 | Atlantinones A | -6.31 | 23.43 | 101516467 |
| 41 | Allantofuranone | -5.55 | 23.48 | 42604569 |
| 42 | β-glucan | -7.48 | 16.07 | 405234154 |
| 43 | Pestalol D | -7.39 | 16.38 | 122389748 |
| 44 | Phomaether A | -7.20 | 16.42 | 139590477 |
| 45 | Usnic Acid | -7.20 | 16.46 | 5646 |
| 46 | Ferulic Acid | -6.84 | 34.70 | 445858 |
| 47 | Gallic Acid | -5.95 | 16.13 | 370 |


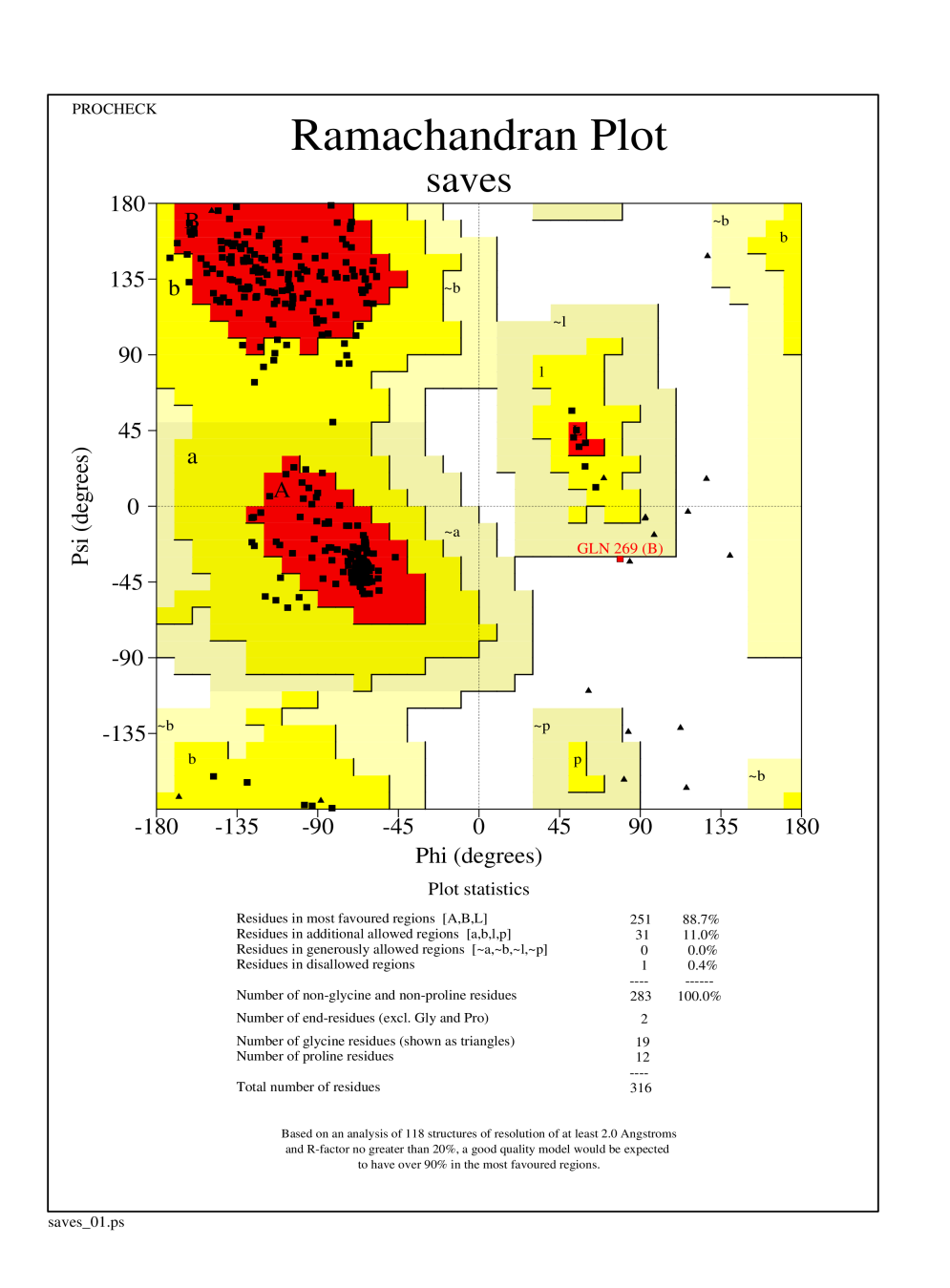


**Figure S1** the Ramachandra plot of PLpro of SARS-CoV-2 showed that 88.7% residues in most favoured regions, 11.0 residues in addition allowed regions and only 0.4% residues in disallowed regions


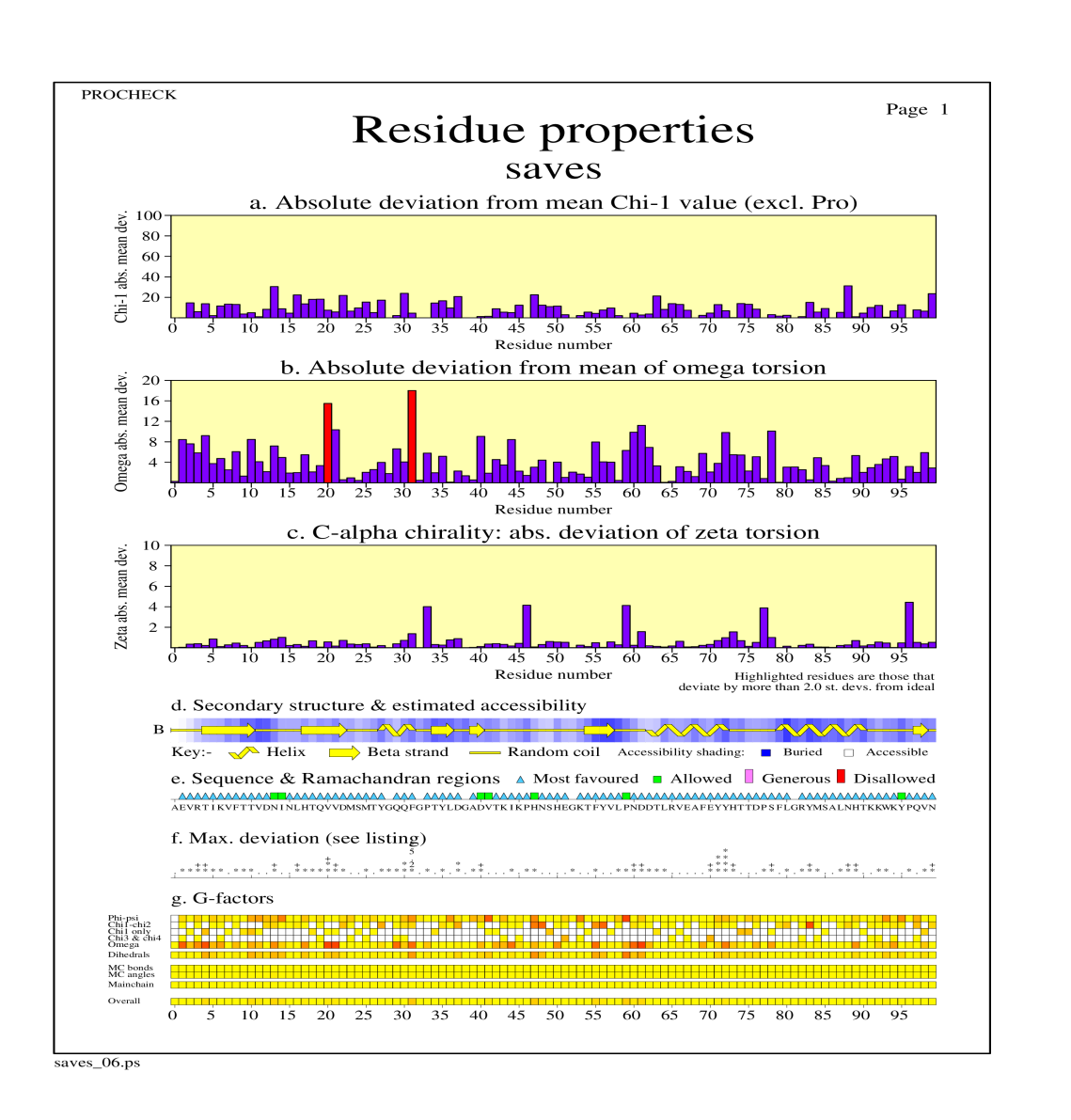


**Figure S2** the residues properties of PLpro of SARS-CoV-2.

**References**

1. Cao X. (2020). COVID-19: immunopathology and its implications for therapy. *Nat Rev Immunol*; 20(5):269-270. Doi: 10.1038/s41577-020-0308-3.
2. World Health Organization (2020). Coronavirus disease 2019 (COVID-19): situation report.
3. Zhou B, Thao TTN, Hoffmann D, *et al*. (2021). SARS-CoV-2 spike D614G changes enhance replication and transmission. *Nature*; 592: 122–127
4. Volz E, Hill V, McCrone J, *et al*. (2021). Evaluating the Effects of SARS-CoV-2 Spike Mutation D614G on Transmissibility and Pathogenicity. *Cell*; 184: 64-75.
5. Mulu A, Gajaa M, Woldekidan HB, and W/mariam JF. (2021). The impact of curcumin derived polyphenols on the structure and flexibility COVID-19 main protease binding pocket: a molecular dynamics simulation study. *PeerJ* 9:e11590
6. Rajpoot S, Alagumuthu M, and S.Baig M. (2021). Dual targeting of 3CLpro and PLpro of SARS-CoV-2: A novel structure-based design approach to treat COVID-19. *Current Research in Structural Biology*; 3: 9-18
7. Fu Z, Huang B, Tang J, Liu S, Liu M, Ye Y, Liu Z, Xiong Y, Zhu W, Cao D, Li X, Niu X, Zhou H, Zhao YJ, Zhang G & Huang H. (2021). The complex structure of GRL0617 and SARS-CoV-2 PLpro reveals a hot spot for antiviral drug discovery. *Nature Communications*; 12: 488
8. Kim KJ, Liu X, Komabayashi T, Jeong SI, and Selli S. (2016). Natural products for infectious diseases Evid. based Compl. *Alternative Med*: eCAM; 9459047
9. Kim JH, Kismali G, and Gupta SC. (2018). Natural products for the prevention and treatment of chronic inflammatory diseases: integrating traditional medicine into modern chronic diseases care Evid. based Compl. Alternative Med; 2018 (2018): 1-10
10. Thomford NE, Senthebane DA, Rowe A, Munro D, Seele P, Maroyi A, and Dzobo K. (2018). Natural products for drug discovery in the 21st century: innovations for novel drug discovery. Int. *J. Mol. Science*; 19 (6): 10
11. Saxena S, Chhibber M, and Pal Singh I. (2019). Fungal Bioactive Compounds in Pharmaceutical Research and Development. *Current Bioactive Compounds*; 15(2): 1-18
12. Biswajit GR. (2017). Potential of small-molecule fungal metabolites in antiviral chemotherapy. *Antiviral Chemistry and Chemotherapy*; 25(2): 20–52.
13. Linnakoski R, Reshamwala D, Veteli P, Cortina-Escribano M, Vanhanen H, and Marjomäki V. (2018). Antiviral Agents From Fungi: Diversity, Mechanisms and Potential Applications. *Front. Microbiol*; 9:2325.
14. Mayer AM, Rodriguez AD, Taglialatela-Scafati O, and Fusetani N. (2013). Marine pharmacology in 2009-2011: marine compounds with antibacterial, antidiabetic, antifungal, anti-inflammatory, antiprotozoal, antituberculosis, and antiviral activities; affecting the immune and nervous systems, and other miscellaneous mechanisms of action. Mar. *Drugs*; 11: 2510–2573.
15. Cheung RC, Wong JH, Pan WL, Chan YS, Yin CM, Dan XL, *et al*. (2014). Antifungal and antiviral products of marine organisms. *Appl. Microbiol. Biotechnol*; 98: 3475–3494.
16. Moghadamtousi ZS, Nikzad S, and Abdul Kadir H. (2015). Potential Antiviral Agents from Marine Fungi: *An Overview. Mar Drugs*; 13: 4520–4538.
17. Butler MS. (2008). Natural products to drugs: natural product-derived compounds in clinical trials. *Nat Prod Rep*; 25: 475–516.
18. Newman DJ, and Cragg GM. (2007). Natural Products as Sources of New Drugs over the Last 25 Years. *J Nat Prod*; 70: 461–477.
19. Rosén J, Gottfries J, and Muresan S. (2017). Novel chemical space exploration via natural products. *J Med Chem*; 52: 1953–1962.
20. Vlasenko V and Vlasenko A. (2018). Antiviral activity of fungi of the Novosibirsk Region: Pleurotus ostreatus and P. pulmonarius (Review). *BIO Web of Conferences*; 11: 00044 (2018).
21. Halgren TA. (1996). Performance of MMFF94. *J. Comput. Chem*; 17: 490–519.
22. Kim S, Thiessen PA, Bolton EE, Chen J, Fu G, Gindulyte A, Han L, He J, He S. Shoemaker BA. (2016). Pub-Chem substance and compound databases. *Nucleic Acids Res*; 44: D1202–D1213.
23. DeLano WL. (2002). The PyMOL Molecular Graphics System, Version 1.1; Schrödinger LLC: New York, NY, USA.
24. Kaplan W, and Littlejohn TG. (2001). Software review Swiss-PDB Viewer (Deep View). *Brief. Bioinform*; 2, 195–197.
25. Daina A, Michielin O, and Zoete V. (2017). SwissADME: A free web tool to evaluate pharmacokinetics, drug-likeness and medicinal chemistry friendliness of small molecules. Sci. Rep; 7: 427-17
26. Cheng F, Li W, Zhou Y, Shen J, Wu Z, Liu G, Lee PW, and Tang Y. (2012). AdmetSAR: A comprehensive source and free tool for assessment of chemical ADMET properties. *J. Chem. Inf. Model*; 52: 3099–3105.
27. Pires DEV, Blundell TL, and Ascher DB. (2015). pkCSM: Predicting small-molecule pharmacokinetic and toxicity properties using graph-based signatures. *J. Med. Chem*; 58: 4066–4072.
28. Lagorce D, Sperandio O, Galons H, Miteva MA, and Villoutreix BO. (2008). FAF-Drugs2: a free ADME/tox filtering tool to assist drug discovery and chemical biology projects. *BMC Bioinformatics*; 9:396
29. Dickson CJ, Madej BD, Skjevik ÅA, Betz RM, Teigen K, Gould IR, and Walker RC. (2014). Lipid14: The amber lipid force field. *J. Chem. Theory Comput*; 10: 865–879.
30. Krieger E, Nielsen JE, Spronk CM, and Vriend G. (2006). Fast empirical pKa prediction by Ewald summation. *J. Mol. Graph. Model*; 25: 481–486.
31. Hosseini M, Chen W, Xiao D, and Wang C. (2021). Computational molecular docking and virtual screening revealed promising SARS-CoV-2 drugs. *Precision Clinical Medicine*; 4(1):1–16.
32. Liu S, Zheng Q, and Wang Z. (2020). Potential covalent drugs targeting the main protease of the SARS-CoV-2 coronavirus. *Bioinformatics*; 36: 3295–8.
33. Rimanshee A, Amit D, Vishal P, *et al*. (2020). Potential inhibitors against papain-like protease of novel coronavirus (SARS-CoV-2) from FDA approved drugs. *ChemRxiv. Preprint*, doi:10.26434/chemrxiv.11860011.v2).
34. E. Allam A, Amen Y, Ashour A, K. Assaf H, Ali Hassan H, Abdel-Rahman IM, M. Sayed A, and Shimizu K. (2021). In silico study of natural compounds from sesame against COVID-19 by targeting Mpro, PLpro and RdRp. *RSC Adv; 11: 22398-22408*
35. Vuong W, Khan MB, Fischer C, Arutyunova E, Lamer T, Shields J, Saffran HA, McKay RT, van Belkum MJ, Joyce MA, Young HS, Tyrrell DL, Vederas JC, and Lemieux MJ. (2020).Feline coronavirus drug inhibits the main protease of SARS-CoV-2 and blocks virus replication. *Nat Commun*; 11(1): 4282.
36. Ma C, Sacco MD, Hurst B, Townsend JA, Hu Y, Szeto T, Zhang X, Tarbet B, Marty MT, Chen Y, Wang J. (2020). Boceprevir, GC-376, and calpain inhibitors II, XII inhibit SARS-CoV-2 viral replication by targeting the viral main protease. *Cell Res*; 30(8):678-692.
37. Kouznetsova VL, Zhang A, Tatineni M, Miller MA, and Tsigelny IF. (2020). Potential COVID-19 papain-like protease PLpro inhibitors: repurposing FDA-approved drugs. PeerJ 8:e9965 DOI 10.7717/peerj.9965
38. Pang J, Gao S, Sun Z, & Yang G. (2021). Discovery of small molecule PLpro inhibitor against COVID-19 using structure-based virtual screening, molecular dynamics simulation, and molecular mechanics/Generalized Born surface area (MM/GBSA) calculation. *Structural Chemistry*; 32: 879–886
39. Sohraby F and Aryapour H. (2021). Unraveling the unbinding pathways of SARS-CoV-2 Papain-like proteinase known inhibitors by Supervised Molecular Dynamics simulation. *PLoS ONE*; 16(5): e0251910. <https://doi.org/10.1371/journal.pone.0251910>
40. Gupta S, Sarthi P, Satyaranjan B, Dipankar S, and Malay Kumar R. (2020): Molecular Mechanism of Clinically Oriented Drug Famotidine with the Identified Potential Target of SARS-CoV-2. ChemRxiv. Preprint. <https://doi.org/10.26434/chemrxiv.12382265.v1>
